# Multi-Transcriptomic Analysis Reveals the Heterogeneity and Tumor-Promoting Role of SPP1/CD44-Mediated Intratumoral Crosstalk in Gastric Cancer

**DOI:** 10.3390/cancers15010164

**Published:** 2022-12-27

**Authors:** Wen Xie, Jia Cheng, Zhijun Hong, Wangyu Cai, Huiqin Zhuo, Jingjing Hou, Lingyun Lin, Xujin Wei, Kang Wang, Xin Chen, Yucheng Song, Zhenfa Wang, Jianchun Cai

**Affiliations:** 1 Department of Gastrointestinal Surgery, Zhongshan Hospital of Xiamen University, School of Medicine, Xiamen University, Xiamen 361001, China; 2 Xiamen Municipal Key Laboratory of Gastrointestinal Oncology, Xiamen 361001, China; 3 The Graduate School of Fujian Medical University, Fuzhou 350004, China

**Keywords:** SPP1, stomach neoplasm, Ming’s classification, CD44, macrophages

## Abstract

**Simple Summary:**

Studies have shown that SPP1 plays an important role in the progression of certain tumors, but the specific functional mechanism in gastric cancer (GC) is still unclear. Our goal was to reveal the prognostic value of SPP1 in GC and its role in the GC tumor microenvironment based on multi-transcriptomic analysis. We demonstrated that a high expression of SPP1 contributed to advanced gastric TNM stages and a poor prognosis for advanced GC patients, providing a solution to the controversy regarding the clinical relevance of SPP1 in GC. Furthermore, we found that SPP1^+^ macrophages were unique to GC cancerous tissues and cancer cell-containing regions, and that the SPP1/CD44-mediated crosstalk between SPP1^+^ macrophages and cancer cells formed a specific niche to accelerate the malignant progression of GC, which may serve as a therapeutic target with fewer side-effects in GC.

**Abstract:**

GC is a fatal disease with high heterogeneity and invasiveness. Recently, SPP1 has been reported to be involved in the tumor progression of multiple human cancers; however, the role of SPP1 in GC heterogeneity and whether it is associated with the invasiveness and mortality of GC remain unclear. Here, we combined multiple RNA sequencing approaches to evaluate the impact of SPP1 on GC. Through bulk RNA sequencing (bulk RNA-seq) and immunohistochemistry (IHC), we found that SPP1 was highly expressed in GC, and high levels of SPP1 were associated with macrophage infiltration, an advanced tumor stage, and higher mortality for advanced GC patients. Furthermore, through simultaneous single-cell and spatial analysis, we demonstrated that SPP1^+^ macrophages are tumor-specific macrophages unique to cancer and enriched in the deep layer of GC tissue. Cell—cell communication analysis revealed that SPP1/CD44 interactions between SPP1^+^ macrophages and their localized tumor epithelial cells could activate downstream target genes in epithelial cells to promote dynamic changes in intratumor heterogeneity. Moreover, these activated genes were found to be closely associated with poor clinical GC outcomes and with cancer-related pathways that promote GC progression, as shown by survival analysis and enrichment analysis, respectively. Collectively, our study reveals that tumor-specific SPP1^+^ macrophages drive the architecture of intratumor heterogeneity to evolve with tumor progression and that SPP1 may serve as a prognostic marker for advanced GC patients, as well as a potential therapeutic target for GC.

## 1. Introduction

GC is a highly invasive disease that remains one of the leading causes of tumor-related deaths, accounting for more than 700,000 deaths annually [1]. Traditionally, GC can be concisely classified into expanding and infiltrative types according to histopathological variants and different growth patterns [2,3], suggesting that GC is a heterogeneous disease in the intertumoral context. Recent studies have shown that intratumor heterogeneity plays a key role in driving tumor evolution, treatment resistance, and disease progression [4,5]; however, for GC, the origins and detailed features of this heterogeneity remain elusive. Therefore, it is necessary to explore the intratumor heterogeneity and related cellular and molecular reprogramming processes in the complex GC ecosystem, which is helpful for elucidating the mechanism of GC development and its progression and for the discovery of potential therapeutic targets.

The complex tumor microenvironment, an important component of intratumor heterogeneity, contains a variety of tissue-resident cells and cells recruited from a distance [6]. Interestingly, the tumor-associated macrophages (TAMs), macrophages present in tumors originating from either circulating monocytes or tissue-resident cells, have multiple phenotypes and function that affect tumor progression [7,8,9]. These heterogeneous TAMs have highly functional plasticity that polarizes into a protumor phenotype in response to environmental stimuli, which in turn impacts tumor cells and other components in the TME [10]. Although there have been reports on the diversity and function of TAMs, the spatial heterogeneity of TAMs and their crosstalk with localized GC cells at high concentrations have not been sufficiently investigated. It is essential to decode the definitive function of TAMs at single-cell and spatial solutions, especially with regard to their role in communicating with other cells to form a niche resulting in tumor progression.

SPP1 is also known as osteopontin and is upregulated in various cancers, including GC; it serves as a multifunctional cytokine involved in various biological processes, such as inflammation, fibrosis, cancer progression, and metastasis [11]. Growing evidence has demonstrated that patients with SPP1 overexpression have a poor prognosis in multiple cancers, including ovarian cancer [12], glioblastoma [13], and melanoma [14]. Regarding GC, however, the prognostic value of SPP1 remains controversial [15,16]. In addition, the expression level of SPP1 in macrophages was higher than that in cancer cells, and macrophage-derived SPP1 could upregulate downstream target genes, leading to tumor progression and drug resistance in cancer cells [17]. It has been reported that SPP1 can bind to various integrins or certain variants of CD44 and activate downstream signaling pathways to promote tumor progression [18]. The above evidence indicates that SPP1 might be a potential therapeutic intervention target for cancer. Nevertheless, the regulation of SPP1 in GC remains unclear. Thus, it is necessary to dig deeper into the prognostic significance of SPP1 and determine the underlying function of SPP1 in GC.

To better understand the expression and biological function of SPP1 in GC, we revealed the presence of a complex tumor ecosystem in GC, in which SPP1^+^ macrophages were enriched in cancerous tissues and cancer cell-containing regions. These findings highlight that SPP1^+^ macrophages are an important component of intratumor heterogeneity. Furthermore, the SPP1/CD44-mediated crosstalk between SPP1^+^ macrophages and cancer cells in the SPP1^+^ macrophage-enriched region formed a specific niche to accelerate the malignant progression of GC, which means that the architecture of intratumor heterogeneity may evolve with tumor progression. In addition, we showed that high expressions of SPP1 contributed to advanced gastric TNM stages and poor prognosis in GC patients. In summary, this study revealed that SPP1 is a poor prognostic indicator for GC patients, and that the SPP1–CD44 axis mediating the interplay between SPP1^+^ macrophages and cancer cells may be a potential therapeutic target for GC.

## 2. Materials and Methods

### 2.1. Bulk RNA-Seq Dataset Retrieval and Analysis

To evaluate the expression levels of selected genes in pan-cancer, we obtained gene expression data in TPM format processed by the Toil [19] pipeline from UCSC XENA (https://xenabrowser.net/datapages/) accessed on 20 March 2022 for TCGA and GTEx samples. Additionally, the same method was used to download the clinical data and gene expression profiles (HTSeq-Counts) for the stomach adenocarcinoma (TCGA-STAD) dataset. According to the diagnostic criteria for GC of Ming’s different growth patterns [20], with the help of pathologists, we selected representative samples of Ming’s expanding and infiltrative types of GC in TCGA (Table S1) and performed differential expression analysis between cancer and normal tissues using the DESeq2 (v1.30.1) package.

We also used CAMOIP [21] (http:/camoip.net/) accessed on 6 August 2022 to evaluate the immune infiltration and pathway enrichment of the TCGA-STAD cohort. With this web tool, we applied the CIBERSORT algorithm to explore the relationship between SPP1 expression and 22 immune cells in GC and used the single-sample gene set enrichment analysis (ssGSEA) method to calculate the pathway scores.

### 2.2. Western Blot

GC and paired adjacent normal tissues were collected and lysed in RIPA buffer (R0010; Solarbio, Beijing, China). We used anti-osteopontin (1:2000 dilution; 22952-1-AP; Proteintech, Wuhan, China) and anti-GAPDH (1:10,000 dilution; AC002; ABclonal, Wuhan, China) as primary antibodies and goat anti-rabbit IgG (H + L) HRP (1:5000 dilution; S0001; Affinity Cincinnati, OH, USA) and goat anti-mouse IgG (H + L) HRP (1:5000 dilution; S0002; Affinity, Cincinnati, OH, USA) as secondary antibodies.

### 2.3. Tissue Microarray (TMA), Clinical Samples, and IHC

One human GC TMA was obtained from Outdo BioTech (Shanghai, China). In addition, we collected 10 GC samples from Zhongshan Hospital, Xiamen University (Xiamen, China) with approval by the medical ethics committee of Zhongshan Hospital, Xiamen University, and the corresponding written informed consent of all patients.

The detailed experimental procedures and immunohistochemical scores were described in our previous study [22]. For this study, we used anti-osteopontin (1:500 dilution; ab214050; Abcam, Cambridge, MA, USA), anti-CD68 (1:10,000 dilution; ab213363; Abcam), and anti-CD44 (1:400 dilution;3570; Cell Signaling Technology, Danvers, MA, USA) as the primary antibodies and calculated the immunohistochemical score based on the staining intensity score multiplied by the staining extent score.

### 2.4. Single-Cell RNA Sequencing (scRNA-seq) Data Acquisition, Processing, and Analysis

We downloaded an scRNA-seq dataset (GSE167297) [23] containing 14 patients with GC from the Gene Expression Omnibus (GEO), used these 14 matrices as the input for Seurat (v4.0.1), and filtered out doublets accessed by Scrublet (v0.2.3). Specifically, cells with a detected gene number of <200 or >5000 or a UMI count of >40,000 were removed, were cells with a high mitochondrial transcript ratio (>20%) or hemoglobin-related transcript ratio (>0.1%). Then, the UMI count data matrix was subjected to the SCTransform pipeline. Harmony (v0.1.0) was used to correct the batch effect between patients. After integration, we used the RunPCA function to perform principal component analysis, and the top 20 PCs were used for RunUMAP and FindNeighbors analyses. Finally, we identified the cell clusters using the FindClusters function with a resolution of 0.6. For scoring the M2 macrophage signature, we applied the AddModuleScore function to calculate the signature scores on the basis of gene sets reported in a previous study [24]. To determine the differentially expressed genes (DEGs), we conducted FindMarkers/FindAllMarkers functions with default parameters.

### 2.5. Cell—Cell Communication

To evaluate the intercellular communications between disparate cell types, we used NicheNet [25] (Nichenetr, v1.0.0) to evaluate the crosstalk and the corresponding target genes between myeloid cells and epithelial cells in different groups (cancer vs. normal; deep vs. superficial). Moreover, we applied CellChat [26] (v1.0.0) to confirm the receptor—ligand pairs, information flow, and interaction strength between myeloid cells and epithelial cells at different depths of invasion in cancerous tissues. Specifically, we used stLearn [27] (v0.4.8) to infer cell—cell communication in spatial transcriptomics.

### 2.6. Pathway Enrichment Analysis

Gene set enrichment analysis (GSEA) was performed using clusterProfiler [28] (v3.18.1) and visualized using GseaVis (v0.0.1) and enrichplot (v1.10.2). In addition, gene set variation analysis (GSVA) was implemented using GSVA (v1.38.2).

### 2.7. Spatial Transcriptomic Data Collection, Quality Control, and Analysis

To explore the spatial information for GC tissues, we obtained a spatial transcriptomic dataset from GEO with the accession number GSE186290 [29] and loaded the outputs produced from Space Ranger (v1.3.1) pipelines into Seurat. Then, we excluded cells with fewer than 500 detected genes and a 1500 UMI count, as well as cells with more than a 5% mitochondrial gene count. For deconvolution, we performed ssGSEA using the top 100 cell-type-specific genes obtained at MCA 2.0 [30] (http://bis.zju.edu.cn/MCA/index.html) accessed on 18 August 2022 to assist in assessing the spatial information of cell types.

### 2.8. Statistical Analysis

Statistical analyses were mostly performed with R software (v4.1.2). For unpaired continuous variables, the Wilcoxon rank-sum test was carried out, whereas, for paired continuous variables, the Wilcoxon sign rank test was employed. Survival analysis determined by a log-rank survival test was conducted with survival (v3.2-10) and survminer (v0.4.9) R packages. For all tests, a *p*-value less than 0.05 was considered statistically significant.

## 3. Results

### 3.1. Expression Pattern of SPP1 in GC Specimens

**Figure 1:**
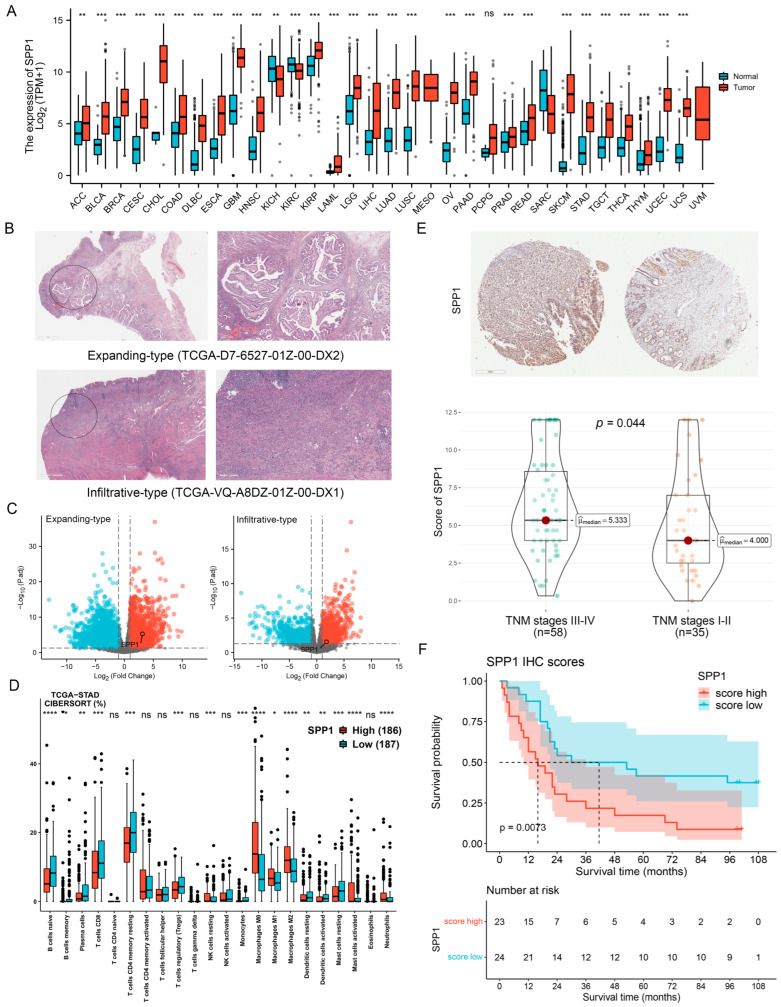
Expression pattern of SPP1 in GC. (**A**) Expression of SPP1 in unpaired samples from XENA-TCGA_GTEx. Wilcoxon rank-sum test. (**B**) HE staining images of expanding-type (top) and infiltrative-type (bottom) GC from TCGA database. Scale bars: 2 mm (left); 400 μm (right). (**C**) Volcano plots showing the DEGs between cancerous and normal tissues in GC with different growth patterns from TCGA. (**D**) Boxplots showing the immune cell scores of the SPP1-High and SPP1-Low expression groups in TCGA. Wilcoxon rank-sum test. (**E**) IHC staining showing SPP1 expression at different stages of the GC TNM staging system. Wilcoxon rank-sum test. Scale bar: 400 μm. (**F**) Kaplan—Meier survival curve showing that advanced GC patients (Stage III) with high expression of SPP1 in GC had a poorer prognosis. ns, *p* ≥ 0.05; * *p* < 0.05; ** *p* < 0.01; *** *p* < 0.001; **** *p* < 0.0001.

We used data from XENA-TCGA_GTEx to investigate SPP1 expression profiles between cancerous and normal tissues in different cancer types. The results revealed that the expression levels of SPP1 were significantly increased in cancerous tissues compared with normal tissues in a variety of cancer types, including GC (Figure 1A and Figure S1A,B). Next, we analyzed SPP1 expression in Ming’s expanding-type and infiltrative-type GC from the TCGA-STAD dataset and found that both types upregulated SPP1 in cancerous tissues (Figure 1B,C). To investigate the potential immune infiltration effect of SPP1 in the TME, the CIBERSORT algorithm was used to estimate the proportions of 22 immune cell types for each sample between the SPP1-High and SPP1-Low groups from TCGA. We observed that the infiltration levels of macrophages were higher in the SPP1-High group than in the SPP1-Low group, while patients in the SPP1-Low group had a significant increase in the fraction of B cells, plasma cells, and CD8^+^ T cells (Figure 1D). Furthermore, we detected the protein level of SPP1 by IHC staining to explore the relationship between SPP1 expression and the TNM staging system and prognosis of patients. As shown in Figure 1E,F, the protein level of SPP1 was higher in patients at TNM stages III–IV, and advanced GC patients with higher expression of SPP1 in GC had poorer prognosis. Then, we compared the ssGSEA scores between the SPP1-High group and the SPP1-Low group in TCGA, which demonstrated that SPP1 might participate in the process of macrophage activation, tumor progression, and metastasis (Figure S1C). These results indicated that high SPP1 expression in GC was associated with a gene expression pattern related to tumor progression, metastasis, and a poor prognosis.

### 3.2. Single-Cell Landscape of GC

**Figure 2:**
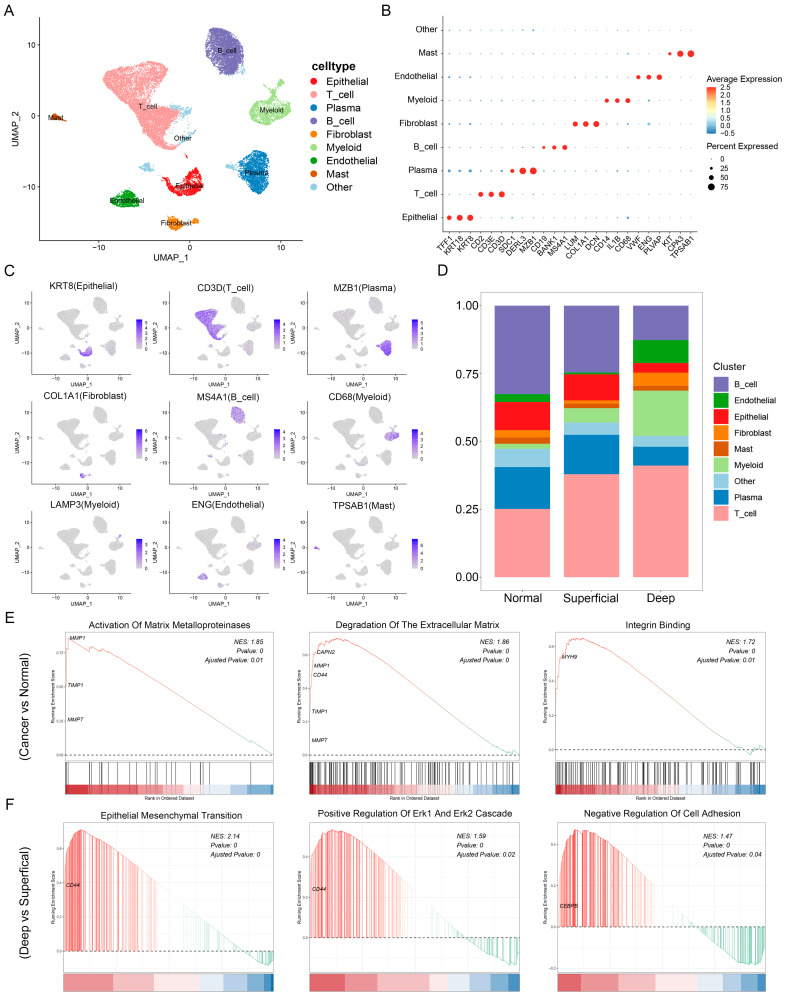
Single-cell landscape of the GC tumor environment. (**A**) UMAP of the GC single-cell landscape annotated by cell type. (**B**) Dot plot showing selected genes for each cell type. Dot size indicates the percentage of expressing cells, colored on the basis of average expression. (**C**) UMAP plots showing the expression of known marker genes. (**D**) Fraction of cell types originating from normal gastric tissues and the superficial and deep GC layers. (**E**) GSEA of the DEGs in epithelial cells between cancerous and normal gastric tissues. (**F**) GSEA of the DEGs in epithelial cells between the deep and superficial GC layers.

To further explore the distribution of SPP1 within GC at the single-cell resolution, the scRNA-seq dataset (GSE167297) was obtained from GEO. After quality control and correction of the batch effects, a total of 22,630 cells originating from cancerous and paired normal tissues of GC were classified into nine clusters (Figure 2A). We annotated the cell types using canonical marker genes: epithelial cells (TFF1, KRT18, and KRT8), T cells (CD2, CD3E, and CD3D), plasma cells (SDC1, DERL3, and MZB1), B cells (CD19, BANK1, and MS4A1), fibroblasts (LUM, COL1A1, and DCN), myeloid cells (CD14, IL1B, and CD68), endothelial cells (VWF, ENG, and PLVAP), and mast cells (KIT, CPA3, and TPSAB1). Additionally, one of the clusters that could not be recognized was labeled “other” (Figure 2B,C). Then, we explored the proportions of each cell cluster among normal gastric tissues and the superficial and deep GC layers. The results revealed that the levels of epithelial cells were lower in the deep layers of cancerous tissues, whereas the numbers of myeloid cells were obviously higher (Figure 2D).

Next, we focused on the functional heterogeneity of epithelial cells among different locations. GSEA revealed the enrichment of the “activation of matrix metalloproteinases”, “degradation of the extracellular matrix”, and “integrin binding” in cancerous tissues as compared to normal gastric tissues (Figure 2E). Likewise, similar observations were made when GSEA was repeated across different GC layers, where signaling to epithelial–mesenchymal transition (EMT) and the MAPK pathway were shown to be enriched in the deep layers of cancerous tissues compared to their superficial counterparts (Figure 2F). The results suggested that cancer cells are more invasive in the deep layers than in the superficial layers of cancerous tissues. Albeit not the predominant cell populations within the deep layers of cancerous tissues, epithelial cells play an important role in intratumoral heterogeneities, and their impact on facilitating tumor progression should not be ignored. This in turn allowed us to focus more on the increased myeloid cells in the deep layers of cancerous tissues. In combination with previous observations that SPP1 is closely related to the infiltration and function of macrophages derived from myeloid cells, we selected myeloid cells for further investigation.

### 3.3. Single-Cell Characterization of Tumor-Infiltrating Myeloid Cells in GC

**Figure 3:**
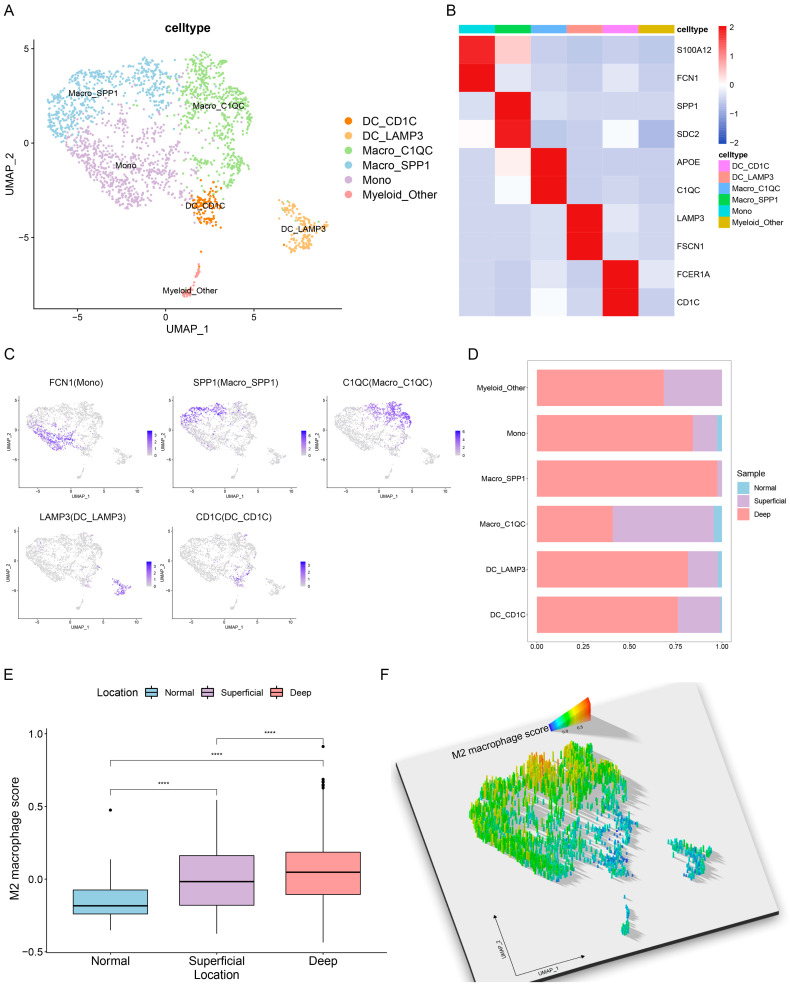
Single-cell characterization of tumor-infiltrating myeloid cells in GC. (**A**) UMAP plot showing the annotated cell types of myeloid cells. (**B**,**C**) Averaged heatmap and UMAP plots showing the expression levels of canonical marker genes across the indicated myeloid clusters. (**D**) Stacked bar plots showing the percentage of major myeloid cell types in different locations. (**E**) Box plot showing the scores of M2 macrophage genes across locations. Wilcoxon rank-sum test. **** *p* < 0.0001. (**F**) The 3D projection of M2 macrophage scores determined by the rayshader R package. Cell type annotations are provided in (**A**).

To investigate the characterization of myeloid cells in the TME of GC, we further subdivided myeloid cells into subpopulations on the basis of canonical markers at a single-cell resolution (Figure 3A–C). The subpopulations were annotated as follows: CD1C^+^ dendritic cells (CD1C and FCER1A), LAMP3^+^ dendritic cells (LAMP3 and FSCN1), C1QC^+^ macrophages (C1QC and APOE), SPP1^+^ macrophages (SPP1 and SDC2), monocytes (S100A12 and FCN1), and other myeloid cells (unidentified). Significantly, we found that the percentage of SPP1^+^ macrophages was highest in the deep GC layer, followed by the superficial GC layer, while it was absent in normal gastric tissues; these results are similar to a previous study on colon cancer [31] (Figure 3D). Additionally, these observations revealed that the tumor-specific SPP1^+^ macrophages that are unique to cancer were an important component of intratumor heterogeneity. In addition, the M2 macrophage scores were calculated with given gene sets for each cell among different locations, and the results showed that cells in the deep GC layer exhibited the highest M2 macrophage score (Figure 3E). We also found that SPP1^+^ macrophages were enriched for the M2 macrophage gene sets (Figure 3F), indicating their tumor-promoting functional phenotype in the GC TME.

### 3.4. Ligand—Receptor Interactions in the TME

**Figure 4:**
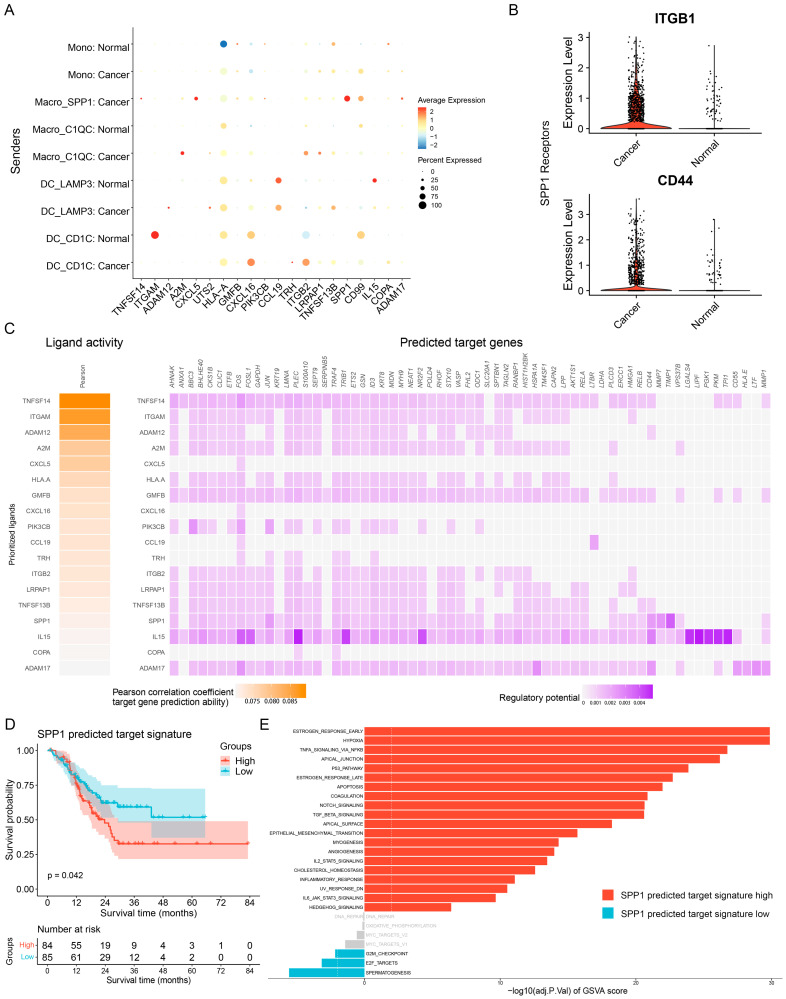
Ligand–receptor interactions between different cell types in cancerous and normal gastric tissue. (**A**,**B**) Inferred ligand—receptor interactions between annotated myeloid cells and epithelial cells in cancerous and normal gastric tissues. Top 20 ligands expressed in annotated myeloid cells (**A**), and SPP1 receptors expressed in epithelial cells (**B**). (**C**) Activated target genes of the top-ranked ligands. (**D**) The Kaplan—Meier overall survival curve of GC patients grouped by the GSVA scores of the predicted SPP1 target signature based on the top and bottom 25% of samples according to the GSVA scores. The *p*-value was calculated by the log-rank test. (**E**) Differences in pathway activities scored by GSVA between the high (*n* = 84 patients) and low (*n* = 85 patients) predicted SPP1 target signatures.

Cell—cell communication between TME cells and cancer cells could contribute to GC heterogeneity via the induction of distinct tumor-promoting functional phenotypes. We then inferred ligand–receptor interactions between myeloid and epithelial cells in cancerous and normal gastric tissues. Notably, as a ligand originating from SPP1^+^ macrophages based on the average expression, SPP1 was one of the most abundant ligands in cancerous tissues, implicating a potential connection between SPP1 expression and modulation of the tumor-supportive microenvironment (Figure 4A). Furthermore, epithelial cells from cancerous tissues showed preferential expression of ITGB1 and CD44 (Figure 4B), which have been reported to be linked to tumor progression [32,33] and could act as a receptor for SPP1 [34]. On the basis of these findings, we next explored the potential target genes affected by SPP1 in cancerous tissues (Figure 4C) and performed functional enrichment analysis of the target genes. Notably, from the GO and KEGG enrichment analysis results, we observed that these target genes contained highly expressed genes related to tumor-promoting pathways, including extracellular matrix disassembly, negative regulation of the apoptotic signaling pathway, and response to hypoxia (Figure S1D). To assess the potential clinical relevance of target genes affected by SPP1, we utilized GSVA to calculate a gene set enrichment score on a per-sample basis of the top 10 target genes (SPP1 predicted target signature) using the STAD dataset from TCGA and classified the STAD samples (the top and bottom 25% of samples according to GSVA scores) into two groups. The survival analysis indicated that the group with a high predicted SPP1 target signature was associated with poor prognosis (Figure 4D), supporting the tumor-promoting characteristics of SPP1. To uncover which pathways were upregulated in the high predicted SPP1 target signature group, a comparison between the two groups revealed a strong enrichment of pathways for the promotion of malignant phenotypes in the high-SPP1 group (Figure 4E).

**Figure 5:**
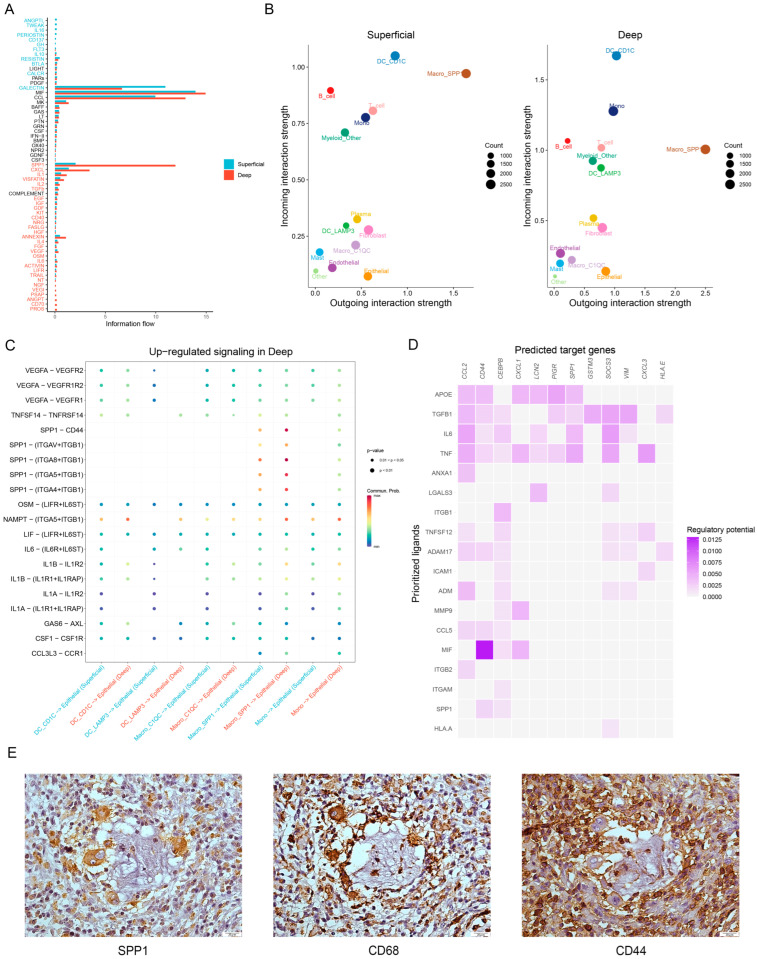
Cell–cell communication between myeloid cells and epithelial cells in the superficial and deep GC layers. (**A**) Overall information flow of each signaling pathway in the different GC layers. The top signaling pathways colored red were enriched in the deep layer, while those colored blue were enriched in the superficial layer. Paired Wilcoxon test. (**B**) Scatter plots showing the dominant sources and targets of signaling between the two groups. (**C**) Dot plot showing the upregulated signaling ligand—receptor pairs between myeloid and epithelial cells in the deep GC layer compared to the superficial GC layer. The color indicates the communication probability. The sizes of the dots indicate the *p*-value of the interactions. (**D**) Inferred target genes activated by the top-ranked ligands from the myeloid cells. (**E**) IHC showing the spatial colocalization of infiltrating SPP1^+^ macrophages and CD44^+^ epithelial cells in the GC specimens. Scale bars, 20 μm.

Next, we sought to investigate whether the intercellular communication between myeloid cells and epithelial cells is different in patients across the different layers of cancerous tissues. We evaluated the information flow per signaling pathway and then ranked the significant pathways on the basis of the differences in each signaling pathway under the total weights in the inferred network. The results showed that SPP1-associated information flow was significantly increased in the deep GC layer (Figure 5A). Similar results were obtained for comparison of the incoming and outcoming interaction strength between different layers of cancerous tissues, in which SPP1^+^ macrophages emerged to be one of the major sources of signals in the deep layer when compared to the superficial layer (Figure 5B). Among the dysregulated signaling ligand—receptor pairs between myeloid and epithelial cells, we identified that SPP1–CD44 was upregulated in the deep GC layer (Figure 5C). Of interest, CD44 and CEBPB were considered the target genes of SPP1 based on comparisons between different locations in cancerous tissues (Figure 5D), and it has been reported that CEBPB is associated with angiogenesis, stemness, distant metastasis, and drug resistance in GC [35,36]. Interestingly, the results of IHC revealed that there was a spatial co-expression pattern of SPP1^+^ macrophages and CD44^+^ epithelial cells in gastric cancer specimens (Figure 5E), providing evidence for the crosstalk between these two cell types.

Taken together, these findings suggested the presence of two major populations in the TME of GC, including SPP1^+^ macrophages and CD44^+^ epithelial cells. The former expressed SPP1 ligand binding to the CD44 receptor of epithelial cells, and the latter presented target genes affected by SPP1, resulting in a protumorigenic phenotype in the TME. The SPP1–CD44 interactions were enriched in cancerous tissues, especially in the deep GC layer, exhibiting an important heterogeneous feature of the GC microenvironment.

### 3.5. Spatial Expression Characteristics of SPP1 Revealed by Spatial Transcriptomics

**Figure 6:**
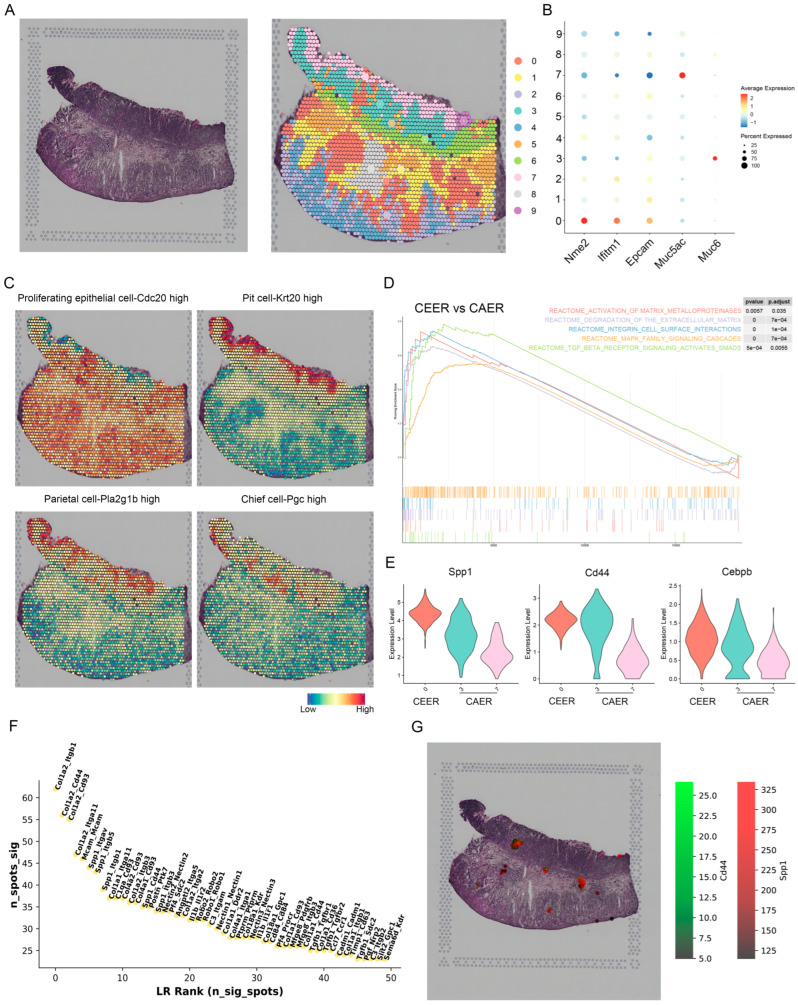
Spatial expression characteristics of SPP1. (**A**) H&E-stained tissue images (left) and spatial transcriptomics cluster map (right) of gastric cancer from mice. (**B**) Dot plot showing selected genes across spatial transcriptomic spots grouped by cluster. (**C**) Spatial feature plots showing the scores of selected cell types from the public database. (**D**) GSEA of the DEGs between the cancer-enriched epithelial region and the cancer-adjacent epithelial region (CEER vs. CAER). (**E**) Violin plots showing the expression of selected genes among Cluster 0, Cluster 3 and Cluster 7. (**F**) Plot showing the overall ranking of ligand—receptor pairs by significant spots. (**G**) Plot showing the spatial positions of the SPP1–CD44 axis. The receptor is in green, and the ligand is in red.

To further unveil the spatial patterns of SPP1 expression in GC, we analyzed spatial transcriptomic data obtained from GSE186290, which contained one tumor tissue section from an orthotopic model of GC in mice. After quality control, a total of 2364 spots were sorted into 10 clusters (Figure 6A). We found that Cluster 0 exhibited a broader and more irregular distribution of cells and highly expressed epithelial markers, including Nme2 and Ifitm1. It has been reported that the two epithelial markers mentioned above are involved in the malignant phenotypic state of GC [37,38], suggesting the malignant characteristics of cells in Cluster 0. However, Cluster 7 and Cluster 3, which were abundant in cancer-adjacent tissues, showed high expressions of Muc5ac and Muc6, respectively (markers of pit mucous cells and gland mucous cells, respectively) (Figure 6B). Since each spot is approximately 50 microns in diameter and may contain 1–10 cells, each cluster represents the gene expression of all spots in the region that may contain different types of cells. On this basis and to further validate our findings, we used the ssGSEA method to score spots by the gene signatures of annotated clusters in single-cell RNA sequencing data of mice. The results showed that Cluster 0 tended to be dominated by proliferative epithelial cells, whereas Clusters 7 and 3 tended to be dominated by pit mucous cells and gland mucous cells (parietal cells and chief cells), respectively (Figure 6C). Specifically, we defined the region where Cluster 0 was located as the cancer-enriched epithelial region (CEER), while the region where Clusters 7 and 3 were located was defined as the cancer-adjacent epithelial region (CAER). The GSEA results showed that genes specifically expressed in the CEER were enriched in cancer-related pathways, including the TGF-beta signaling pathway, MAPK signaling pathway, EMT-related pathways, and integrin cell surface interactions (Figure 6D); notably, these results are similar to the findings discussed in the previous sections. We also assessed the expression levels of Spp1, Cd44, and Cebpb among different epithelial regions and found that these genes were more strongly expressed in the CEER than in the CAER (Figure 6E). This indicated that these genes had a propensity for distribution to cancerous regions. As shown in Figure 6F, the Spp1–Cd44 axis was identified as one of the top-ranking ligand—receptor pairs according to the overall ranking of ligand—receptor pairs based on the significant spots calculated by stLearn. Moreover, Spp1–Cd44 interactions were also enriched in the CEER (Figure 6G), thus forming a cancer-promoting crosstalk that resulted in the invasiveness of epithelial cells. This result indicated that the spatial location and localized microenvironment of cells were part of the reason for intratumor heterogeneity.

## 4. Discussion

In this study, we analyzed different types of transcriptome sequencing data to comprehensively explore the expression pattern of SPP1 in GC. The results showed that SPP1 expression was closely related to the advanced stage of cancer and poor prognosis of patients with advanced GC. Furthermore, we found that tumor-specific SPP1^+^ macrophages were a crucial component of intratumor heterogeneity, and that SPP1/CD44-mediated crosstalk played an important role in GC progression. Our study revealed underlying mechanisms for the formation of intratumor heterogeneity by exerting diverse selective pressures in different regions of the TME. These findings can provide a valuable reference for further exploration to obtain detailed biological functions and develop new immune checkpoints or therapeutic targets.

In the present study, we demonstrated that SPP1 was widely upregulated in multiple cancerous tissues, including GC, and it was associated with advanced gastric cancer stages (AJCC TNM stage III–IV). In addition, it could be a prognostic factor for a poor GC prognosis based on the results of bulk RNA-seq data and IHC in tissue samples. In line with our findings, others have shown that the expression of SPP1 was elevated in a variety of cancerous tissues compared to normal tissues (GC, lung cancer, hepatocellular carcinoma, etc.). Even with the agreement that SPP1 was upregulated in GC, previous studies yielded conflicting observations regarding the prognostic role of SPP1 in GC. Several studies evaluating SPP1 expression from microarray data have suggested that there is no correlation between SPP1 expression in GC and the poor prognosis of patients [16,39]. In contrast, Bartolomeo et al. showed that higher SPP1 expression was related to a poorer prognosis according to the IHC results of GC patients [15], which is consistent with our findings in this study. The different approaches used to assess the expression of SPP1 may account for the discrepancies in these studies. The observations of Bartolomeo et al. and our study were made at the protein level, while the other observations mentioned above were made at the transcriptomic level. Notably, our findings are consistent with the observations in the Bartolomeo et al. study, while also extending them. We found that SPP1 expression was significantly increased in advanced-stage gastric cancer (AJCC TNM stages III–IV) compared with early-stage gastric cancer (AJCC TNM stages I-II) in our GC cohorts. Considering the tumor heterogeneity of gastric cancer, this novel finding may result from the intertumor heterogeneity of patients between our cohort and the cohort of Bartolomeo et al. Interestingly, the AJCC TNM staging systems of malignant tumors are essential in assessing the strategy of adjuvant chemotherapy, and we conjecture that SPP1 may have the potential to be a predictor participating in the selection of adjuvant chemotherapy strategies in GC. However, this field requires investigation in future studies. Briefly, our findings further suggest that SPP1 may serve as an auxiliary biomarker for the qualitative assessment of disease severity and help to identify the clinical outcomes of gastric cancer patients.

Tumor-associated macrophages are a common component of the TME, and they have complex classifications and diverse biological functions [7]. Although binary classification does not adequately reflect the compositional and functional complexity of TAMs, the polarization state of TAMs can be roughly divided into M1 and M2 phenotypes, which exert antitumor and tumor-promoting roles, respectively [40]. One pan-cancer study showed that SPP1^+^ TAMs with the M2 phenotype were found in several cancers but not in GC [24]. However, Jeong et al. found that SPP1 was predominantly expressed in myeloid cells and that SPP1-enriched myeloid cells were distributed only in the deep layers of GC tissues by IHC. Importantly, on the basis of scRNA-seq data derived from the Jeong et al. cohort, we identified that SPP1^+^ macrophages exhibiting the M2 macrophage phenotype were found only in cancerous tissues and mainly in the deep layers of these cancerous tissues by elaborately subsetting and reclustering myeloid cells. Moreover, we further validated our findings by IHC of SPP1 and CD68, which are commonly used as macrophage markers for histological analysis [41,42,43]. Similar to our findings, Zhang et al. reported that SPP1^+^ TAMs were essentially absent in colorectal noncancer tissues [31]. The different observations between our study and the pan-cancer cohort may be due to different sample collection designs, starting populations and intersample diversity. Moreover, one possible reason for the discrepancies in the distribution of SPP1^+^ macrophages between our study and the Jeong et al. cohort may be the different analysis methods for assessing the distributions; they used the IHC results to evaluate the distribution of SPP1, while we capitalized on the bioinformatics findings. Beyond defining SPP1^+^ macrophages and their distribution, our work goes one step further than previous studies to provide evidence for the cancerous region-specific distribution of this kind of macrophage according to the comparison of SPP1 expression between CEER and CAER at a spatial resolution. Consequently, we speculate that SPP1 may be a potential target for GC treatment due to its cancerous region-specific distribution, which may reduce the side-effects on normal cells. Notably, additional work will be needed to test this hypothesis.

The remodeling of the TME and the complex crosstalk among the various components of the TME play an important role in the process of tumorigenesis and progression [44]. In this study, we reported that intercellular communication between SPP1^+^ macrophages and epithelial cells via the SPP1–CD44 axis was exclusively enhanced in the GC tissues, especially in the deep layers of these tissues. Consistently, as shown in the spatial transcriptomics, the SPP1–CD44 axis had a clear propensity for the distribution of cancer regions. Furthermore, these enhanced communications activated downstream target genes that were involved in cancer-related pathways, including the TGF-beta signaling pathway, MAPK signaling pathway, EMT-related pathways, and integrin cell surface interactions, leading to tumor progression and poor clinical GC outcomes. Liu et al. showed that the SPP1–CD44 axis in hepatocellular carcinoma (HCC) leads to tumor progression, and that this axis could not be identified in the tumor-adjacent tissues of HCC, which is similar to the findings of our present study [45]. Recently, an in vitro study confirmed our findings regarding the role of the SPP1–CD44 axis in the tumorigenesis function of prostate cancer and found downstream pathways to promote tumor progression [46]. Although the protumor role of the SPP1–CD44 axis in the interaction between macrophages and cancer cells has been investigated for several cancers, its role in gastric cancer has not been reported. In addition, QI et al. suggested that the crosstalk between SPP1^+^ macrophages and FAP^+^ fibroblasts in colon cancer served as a potential therapeutic target for improving the efficacy of immunotherapy through combined single-cell and spatial analysis [10]. Similarly, our findings may support the underlying mechanisms regarding the hypothesis that disrupting the crosstalk between SPP1^+^ macrophages and CD44^+^ epithelial cells could be a potential strategy for GC treatment.

However, several limitations are worth noting in the current study. The first limitation is the quantity of samples, including one bulk RNA-seq cohort of STAD from TCGA, one scRNA-seq cohort consisting of 14 GC samples, and one spatial transcriptomic cohort containing a tumor section from a mouse GC model. This small number of samples may not have sufficient power to obtain cells for covering the histologic features of all patients and identifying all cell clusters in GC. Therefore, more research with a larger sample size may help to clarify this issue. The second limitation is that the scRNA-seq cohort based on diffuse-type gastric cancers may not represent the expression characteristics of other subtypes of GC, and further studies should be conducted to explore whether these observations are relevant in other GC subtypes. The third limitation is the lack of relevant functional validation in vivo and in vitro. Further functional experiments in vivo and in vitro may validate our findings in the future.

## 5. Conclusions

Taken together, our study sheds light on the intratumor heterogeneity mediated by the expression pattern of SPP1 and the distribution of tumor-specific SPP1^+^ macrophages, as well as the cancer region-specific SPP1–CD44 axis. Activation of the SPP1–CD44 axis between TAMs and cancer cells in localized regions within cancerous tissues subsequently triggers cancer-related signaling pathways, leading to exacerbation of the malignant phenotype in the related regions under locally selective pressures. This may serve as the foundation for the exploration of new therapeutic strategies and targets in patients with GC.

## Data Availability

Datasets used in current study are available in the GEO, TCGA, and the GTEx databases. All codes generated during this study are available from the corresponding author on reasonable request.

## Supplementary Materials

The following supporting information can be downloaded at https://www.mdpi.com/article/10.3390/cancers15010164/s1. Figure S1. SPP1 expression and functional enrichment of SPP1-related genes; Table S1. Representative samples of expanding and infiltrative types of GC in TCGA.
