# Supplementary material for: Multi-Transcriptomic Analysis Reveals the Heterogeneity and Tumor-Promoting Role of SPP1/CD44-Mediated Intratumoral Crosstalk in Gastric Cancer
Source: Cancers (Basel). 2022 Dec 27;15(1):164. doi: 10.3390/cancers15010164 (PMC9818284; doi:10.3390/cancers15010164)
Supplement: Supplementary file 1 [file cancers-15-00164-s001.zip › FigureS1.final.pdf]

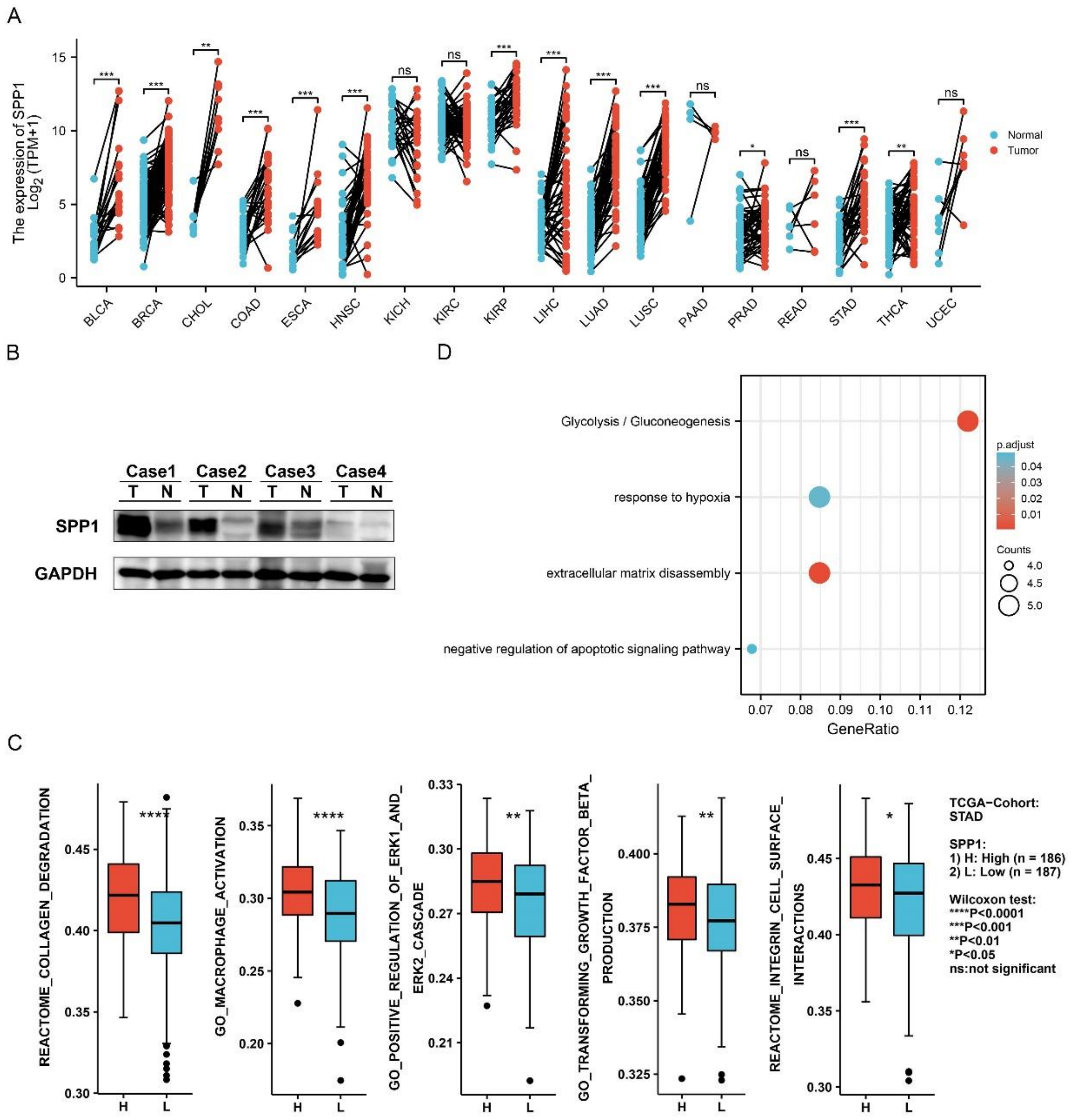

Figure S1. SPP1 expression and functional enrichment of SPP1-related genes

(A) Expression of SPP1 in paired samples from XENA-TCGA_GTEx. Wilcoxon rank-sum test. **(B)** Western blot showing the protein levels of SPP1 between GC and paired adjacent normal tissues. T, Tumor; N, Normal. **(C)** Bar plots showing the functional enrichment between the SPP1-High and SPP1-Low groups. **(D)** Dot plot showing the GO and KEGG analysis results of SPP1-predicted target genes.
